# Supplementary material for: Differential Hatching, Development, Oviposition, and Longevity Patterns among Colombian Aedes aegypti Populations
Source: Insects. 2022 Jun 10;13(6):536. doi: 10.3390/insects13060536 (PMC9224916; doi:10.3390/insects13060536)
Supplement: Supplementary file 1 [file insects-13-00536-s001.zip › SupplementaryMaterial.pdf]

Supplementary Material

**Differential hatching, development, oviposition, and longevity patterns among Colombian *Aedes aegypti* populations**

**Andrea Arévalo-Cortés[1*], Yurany Granada[1], David Torres[1], Omar Triana-Chavez[1].**

**Table S1.** Life cycle (days) from egg to adult (male and female) of Colombian *Ae. aegypti* from different regions reared at 28±1°C temperature, 80±5% relative humidity, and 12 h light: 12 h dark photoperiod. Minimum (Min); Maximum (Max); Standard deviation (SD). Pupation time is the mean development time from L4 to pupae. Time of emergence is the mean development time from pupae to adults (male or female).

| | Bello | | | Neiva | | | Itagüí | | | Riohacha | | | Rockefeller | | |
|---|---|---|---|---|---|---|---|---|---|---|---|---|---|---|---|
| Development parameters | Min | Max | Mean±SD | Min | Max | Mean±SD | Min | Max | Mean±SD | Min | Max | Mean±SD | Min | Max | Mean±SD |
| Egg to L1 (Eclosion) | 1.0 | 10.0 | 1.22 ± 0.61 | 1.0 | 10.0 | 1.39 ± 1.49 | 1.0 | 21.0 | 1.93 ± 1.81 | 1.0 | 25.0 | 2.49 ± 3.3 | 1.0 | 21.0 | 2.19 ± 2.33 |
| L1+L2 to L3 | 2.0 | 11.0 | 2.09 ± 0.63 | 2.0 | 10.0 | 3.13 ± 1.43 | 2.0 | 10.0 | 3.65 ± 1.83 | 3.0 | 27.0 | 6.26 ± 3.05 | 2.0 | 25.0 | 4.27 ± 2.70 |
| L3 to L4 | 1.0 | 13.0 | 1.14 ± 0.87 | 1.0 | 10.0 | 2.64 ± 1.57 | 1.0 | 12.0 | 3.25 ± 1.84 | 1.0 | 25.0 | 4.14 ± 3.04 | 1.0 | 26.0 | 3.04 ± 2.41 |
| L1+L2 to L4 (Total larval development time) | 3.0 | 16.0 | 3.17 ± 1.03 | 3.0 | 20.0 | 5.70 ± 2.87 | 3.0 | 21.0 | 6.84 ± 3.53 | 5.0 | 52.0 | 10.40 ± 6.03 | 3.0 | 42.0 | 7.18 ± 4.64 |
| L4 to Pupae (Pupation time) | 2.0 | 11.0 | 3.11 ± 0.73 | 2.0 | 10.0 | 6.49 ± 1.49 | 2.0 | 26.0 | 5.25 ± 2.02 | 1.0 | 24.0 | 4.38 ± 2.99 | 0.0 | 19.0 | 3.90 ± 2.03 |
| Pupae to Male (Male emergence time) | 2.0 | 5.0 | 3.23 ± 0.53 | 1.0 | 11.0 | 5.46 ± 1.86 | 2.0 | 10.0 | 4.46 ± 1.83 | 1.0 | 25.0 | 5.35 ± 3.37 | 2.0 | 10.0 | 4.16 ± 1.93 |
| Pupae to Female (Female emergence time) | 3.0 | 12.0 | 3.81 ± 0.98 | 2.0 | 11.0 | 6.41 ± 1.63 | 2.0 | 10.0 | 5.24 ± 1.59 | 2.0 | 25.0 | 5.36 ± 2.48 | 2.0 | 21.0 | 5.09 ± 2.32 |
| Average age Egg to Male | 8.0 | 50.0 | 10.79 ± 3.37 | 7.0 | 51.0 | 19.11 ± 7.84 | 8.0 | 79.0 | 18.54 ± 9.33 | 7.0 | 126.0 | 22.62 ± 15.75 | 6.0 | 101.0 | 17.56 ± 11.4 |
| Average age Egg to Female | 9.0 | 57.0 | 11.37 ± 3.82 | 8.0 | 51.0 | 20.06 ± 7.61 | 8.0 | 79.0 | 19.32 ± 9.09 | 8.0 | 126.0 | 22.63 ± 14.86 | 6.0 | 112.0 | 18.49 ± 11.79 |

**Table S2.** Age specific life table of 100 cohort eggs in each *Ae. aegypti* population. Mosquitoes were reared at 28±1°C temperature, 80±5% relative humidity, and 12 h light: 12 h dark photoperiod. Mean ± standard deviation (SD) of four to six replicates are shown. One way analysis of variance (ANOVA) was conducted followed by a Bonferroni post- hoc test to account for multiple comparisons between *Ae. aegypti* populations. Means in the same row followed by the same letters are not significantly different ($p > 0.05$). Proportion of age-specific survivorship: $l_x$. Proportion of individuals who die during the age interval ($x$) to ($x + 1$): $d_x$. Age-specific mortality rate: $q_x$. Killing power: $k_x$. Average of the probability of survival between two successive ages: $L_x$. Total number of days remaining to live for survivors of the cohort, when it reaches age $x$, until the last member dies at age $m$: $T_x$. Life expectancy, i.e., the mean number of days remaining to the survivors at age $x$: $e_x$.

| Parameters | Developmental stage (*x*) | Bello | Neiva | Itagüí | Riohacha | Rockefeller |
|---|---|---|---|---|---|---|
| *lx* | Egg | 1.00 ± 0.00$^{a}$ | 1.00 ± 0.00$^{a}$ | 1.00 ± 0.00$^{a}$ | 1.00 ± 0.00$^{a}$ | 1.00 ± 0.00$^{a}$ |
| | L1+L2 | 0.988 ± 0.015$^{a}$ | 0.898 ± 0.022$^{b}$ | 0.968 ± 0.033$^{a}$ | 0.984 ± 0.009$^{a}$ | 0.988 ± 0.15$^{a}$ |
| | L3 | 0.960 ± 0.034$^{a}$ | 0.883 ± 0.035$^{b}$ | 0.938 ± 0.04$^{ab}$ | 0.974 ± 0.017$^{a}$ | 0.963 ± 0.021$^{a}$ |
| | L4 | 0.935 ± 0.051$^{ab}$ | 0.865 ± 0.021$^{a}$ | 0.924 ± 0.044$^{ab}$ | 0.974 ± 0.017$^{b}$ | 0.950 ± 0.03$^{b}$ |
| | Pupae | 0.903 ± 0.052$^{ab}$ | 0.855 ± 0.017$^{a}$ | 0.914 ± 0.55$^{ab}$ | 0.966 ± 0.019$^{b}$ | 0.940 ± 0.028$^{b}$ |
| | Adult | 0.848 ± 0.017$^{a}$ | 0.843 ± 0.017$^{a}$ | 0.898 ± 0.051$^{ac}$ | 0.962 ± 0.022$^{b}$ | 0.940 ± 0.028$^{bc}$ |
| *dx* | Egg | 0.013 ± 0.015$^{a}$ | 0.103 ± 0.022$^{b}$ | 0.032 ± 0.033$^{a}$ | 0.016 ± 0.009$^{a}$ | 0.012 ± 0.015$^{a}$ |
| | L1+L2 | 0.028 ± 0.021$^{a}$ | 0.015 ± 0.013$^{a}$ | 0.03 ± 0.028$^{a}$ | 0.01 ± 0.01$^{a}$ | 0.025 ± 0.019$^{a}$ |
| | L3 | 0.03 ± 0.03$^{a}$ | 0.018 ± 0.022$^{a}$ | 0.014 ± 0.013$^{a}$ | 0.00 ± 0.00$^{a}$ | 0.013 ± 0.012$^{a}$ |
| | L4 | 0.033 ± 0.025$^{a}$ | 0.01 ± 0.008$^{a}$ | 0.01 ± 0.017$^{a}$ | 0.008 ± 0.008$^{a}$ | 0.01 ± 0.009$^{a}$ |
| | Pupae | 0.055 ± 0.065$^{a}$ | 0.013 ± 0.013$^{ab}$ | 0.016 ± 0.011$^{ab}$ | 0.004 ± 0.006$^{ab}$ | 0.00 ± 0.00$^{b}$ |
| | Adult | 0.848 ± 0.017$^{a}$ | 0.843 ± 0.017$^{a}$ | 0.898 ± 0.051$^{ac}$ | 0.962 ± 0.022$^{b}$ | 0.940 ± 0.028$^{bc}$ |
| *qx* | Egg | 0.013 ± 0.015$^{a}$ | 0.103 ± 0.022$^{b}$ | 0.032 ± 0.033$^{a}$ | 0.016 ± 0.009$^{a}$ | 0.012 ± 0.015$^{a}$ |
| | L1+L2 | 0.028 ± 0.021$^{a}$ | 0.015 ± 0.013$^{a}$ | 0.03 ± 0.028$^{a}$ | 0.01 ± 0.01$^{a}$ | 0.025 ± 0.02$^{a}$ |
| | L3 | 0.025 ± 0.025$^{a}$ | 0.018 ± 0.022$^{a}$ | 0.014 ± 0.013$^{a}$ | 0.00 ± 0.00$^{a}$ | 0.013 ± 0.012$^{a}$ |
| | L4 | 0.033 ± 0.025$^{a}$ | 0.01 ± 0.008$^{a}$ | 0.012 ± 0.022$^{a}$ | 0.008 ± 0.008$^{a}$ | 0.01 ± 0.009$^{a}$ |
| | Pupae | 0.058 ± 0.064$^{a}$ | 0.013 ± 0.013$^{ab}$ | 0.016 ± 0.011$^{ab}$ | 0.004 ± 0.006$^{ab}$ | 0.00 ± 0.00$^{b}$ |
| | Adult | 1.00 ± 0.00$^{a}$ | 1.00 ± 0.00$^{a}$ | 1.00 ± 0.00$^{a}$ | 1.00 ± 0.00$^{a}$ | 1.00 ± 0.00$^{a}$ |
| *kx* | Egg | 0.005 ± 0.006$^{a}$ | 0.05 ± 0.009$^{b}$ | 0.014 ± 0.017$^{a}$ | 0.008 ± 0.005$^{a}$ | 0.003 ± 0.005$^{a}$ |
| | L1+L2 | 0.01 ± 0.008$^{a}$ | 0.008 ± 0.009$^{a}$ | 0.014 ± 0.015$^{a}$ | 0.004 ± 0.006$^{a}$ | 0.010 ± 0.011$^{a}$ |
| | L3 | 0.013 ± 0.013$^{a}$ | 0.008 ± 0.009$^{a}$ | 0.006 ± 0.006$^{a}$ | 0.00 ± 0.00$^{a}$ | 0.005 ± 0.005$^{a}$ |
| | L4 | 0.015 ± 0.01$^{a}$ | 0.008 ± 0.005$^{a}$ | 0.004 ± 0.009$^{a}$ | 0.002 ± 0.005$^{a}$ | 0.003 ± 0.005$^{a}$ |
| | Pupae | 0.03 ± 0.031$^{a}$ | 0.01 ± 0.008$^{a}$ | 0.006 ± 0.006$^{a}$ | 0.00 ± 0.00$^{a}$ | 0.00 ± 0.00$^{a}$ |
| | Adult | - | - | - | - | - |
| *Lx* | Egg | 0.995 ± 0.006$^{a}$ | 0.953 ± 0.009$^{b}$ | 0.986 ± 0.017$^{a}$ | 0.992 ± 0.005$^{a}$ | 0.997 ± 0.005$^{a}$ |
| | L1+L2 | 0.978 ± 0.022$^{a}$ | 0.893 ± 0.028$^{b}$ | 0.954 ± 0.032$^{a}$ | 0.980 ± 0.012$^{a}$ | 0.978 ± 0.015$^{a}$ |
| | L3 | 0.95 ± 0.041$^{a}$ | 0.878 ± 0.029$^{b}$ | 0.932 ± 0.040$^{ab}$ | 0.974 ± 0.017$^{a}$ | 0.96 ± 0.024$^{a}$ |
| | L4 | 0.92 ± 0.05$^{ab}$ | 0.863 ± 0.017$^{a}$ | 0.92 ± 0.05$^{ab}$ | 0.972 ± 0.016$^{b}$ | 0.95 ± 0.027$^{b}$ |
| | Pupae | 0.88 ± 0.024$^{ac}$ | 0.853 ± 0.015$^{a}$ | 0.908 ± 0.052$^{ab}$ | 0.966 ± 0.019$^{b}$ | 0.94 ± 0.028$^{bc}$ |
| | Adult | 0.428 ± 0.009$^{a}$ | 0.423 ± 0.009$^{a}$ | 0.452 ± 0.028$^{ab}$ | 0.484 ± 0.009$^{b}$ | 0.472 ± 0.016$^{b}$ |
| *Tx* | Egg | 5.133 ± 0.137$^{ab}$ | 4.843 ± 0.104$^{a}$ | 5.142 ± 0.213$^{b}$ | 5.36 ± 0.081$^{b}$ | 5.282 ± 0.106$^{b}$ |
| | L1+L2 | 4.14 ± 0.129$^{ab}$ | 3.898 ± 0.092$^{a}$ | 4.160 ± 0.199$^{ab}$ | 4.368 ± 0.077$^{b}$ | 4.290 ± 0.103$^{b}$ |
| | L3 | 3.17 ± 0.107$^{ab}$ | 3.01 ± 0.07$^{a}$ | 3.21 ± 0.166$^{ab}$ | 3.390 ± 0.064$^{b}$ | 3.313 ± 0.094$^{b}$ |
| | L4 | 2.22 ± 0.066$^{ac}$ | 2.133 ± 0.043$^{a}$ | 2.28 ± 0.128$^{ab}$ | 2.42 ± 0.048$^{b}$ | 2.36 ± 0.068$^{bc}$ |
| | Pupae | 1.30 ± 0.018$^{a}$ | 1.273 ± 0.022$^{a}$ | 1.36 ± 0.079$^{ab}$ | 1.446 ± 0.031$^{b}$ | 1.412 ± 0.044$^{b}$ |
| | Adult | 0.428 ± 0.009$^{a}$ | 0.423 ± 0.009$^{a}$ | 0.452 ± 0.028$^{ab}$ | 0.484 ± 0.009$^{b}$ | 0.472 ± 0.016$^{b}$ |
| *ex* | Egg | 5.133 ± 0.137$^{ab}$ | 4.843 ± 0.104$^{a}$ | 5.142 ± 0.213$^{b}$ | 5.360 ± 0.081$^{b}$ | 5.282 ± 0.106$^{b}$ |
| | L1+L2 | 4.190 ± 0.082$^{a}$ | 4.343 ± 0.022$^{ab}$ | 4.296 ± 0.114$^{ab}$ | 4.440 ± 0.039$^{b}$ | 4.338 ± 0.105$^{ab}$ |
| | L3 | 3.298 ± 0.043$^{a}$ | 3.41 ± 0.065$^{ab}$ | 3.418 ± 0.068$^{b}$ | 3.48 ± 0.019$^{b}$ | 3.44 ± 0.043$^{b}$ |
| | L4 | 2.372 ± 0.057$^{a}$ | 2.463 ± 0.031$^{b}$ | 2.462 ± 0.031$^{b}$ | 2.480 ± 0.019$^{b}$ | 2.480 ± 0.018$^{b}$ |
| | Pupae | 1.44 ± 0.064$^{a}$ | 1.488 ± 0.013$^{ab}$ | 1.484 ± 0.011$^{ab}$ | 1.496 ± 0.005$^{ab}$ | 1.50 ± 0.00$^{b}$ |
| | Adult | 0.50 ± 0.00$^{a}$ | 0.50 ± 0.00$^{a}$ | 0.50 ± 0.00$^{a}$ | 0.50 ± 0.00$^{a}$ | 0.50 ± 0.00$^{a}$ |

**Table S3.** Sex frequency and ratio in *Ae. aegypti* populations reared at 28±1°C temperature, 80±5% relative humidity, and 12 h light: 12 h dark photoperiod. One way analysis of variance (ANOVA) was conducted followed by a Bonferroni post- hoc test to account for multiple comparisons between populations of *Ae. aegypti*. Additionally, a Student's t-test was used to compare female and male percentages within the same population. * $p < 0.05$; ns, no significant difference ($p > 0.05$); SD, standard deviation.

| | Bello | | Neiva | | Itagüí | | Riohacha | | Rockefeller | |
|---|---|---|---|---|---|---|---|---|---|---|
| | Female | Male | Female | Male | Female | Male | Female | Male | Female | Male |
| Frequency (*Ni*) | 136 | 203 | 158 | 179 | 226 | 223 | 221 | 260 | 282 | 282 |
| Percentage Mean ± SD | 40.13 ± 3.61* | 59.88 ± 3.61* | 46.80 ± 6.66$^{ns}$ | 53.2 ± 6.66$^{ns}$ | 50.24 ± 3.48$^{ns}$ | 49.76 ± 3.48$^{ns}$ | 45.88 ± 4.75* | 54.12 ± 4.75* | 49.98 ± 9.94$^{ns}$ | 50.02 ± 9.94$^{ns}$ |
| Sex ratio (female / male) Mean ± SD | 0.67 ± 0.10 $^{ns}$ | | 0.90 ± 0.23 $^{ns}$ | | 1.02 ± 0.14 $^{ns}$ | | 0.86 ± 0.17 $^{ns}$ | | 1.07 ± 0.43 $^{ns}$ | |

**Table S4.** Male life tables of *Ae. aegypti* populations. Mosquitoes were reared at 28±1°C temperature, 80±5% relative humidity, and 12 h light: 12 h dark photoperiod. Mean ± standard deviation (SD) of four to six replicates are shown. Proportion of age-specific survivorship: *lx*. Proportion of individuals who die during the age interval (*x*) to (*x + 1*): *dx*. Age-specific mortality rate: *qx*. Killing power: *kx*. Average of the probability of survival between two successive ages: *Lx*. Total number of days remaining to live for survivors of the cohort, when it reaches age *x*, until the last member dies at age *m*: *Tx*. Life expectancy, i.e., the mean number of days remaining to the survivors at age *x*: *ex*.

**Table S5.** Female life tables of *Ae. aegypti* populations. Mosquitoes were reared at 28±1°C temperature, 80±5% relative humidity, and 12 h light: 12 h dark photoperiod. Mean ± standard deviation (SD) of four to six replicates are shown. Proportion of age-specific survivorship: *lx*. Proportion of individuals who die during the age interval (*x*) to (*x + 1*): *dx*. Age-specific mortality rate: *qx*. Killing power: *kx*. Average of the probability of survival between two successive ages: *Lx*. Total number of days remaining to live for survivors of the cohort, when it reaches age *x*, until the last member dies at age *m*: *Tx*. Life expectancy, i.e., the mean number of days remaining to the survivors at age *x*: *ex*.

**Table S6.** Adult longevity for both sexes in *Ae. aegypti* populations reared at 28±1°C temperature, 80±5% relative humidity, and 12 h light: 12 h dark photoperiod. One way analysis of variance (ANOVA) was conducted followed by a Bonferroni post- hoc test to account for multiple comparisons between populations of *Ae. aegypti*. Mean days ± standard error (SE) are shown. Means in the same row by sex, followed by the same letters are not significantly different ($p > 0.05$). Additionally, a Student's t-test was used to compare female and male means within the same population. * $p < 0.05$.

| Females | | | | | Males | | | | |
|---|---|---|---|---|---|---|---|---|---|
| Bello | Neiva | Itagüí | Riohacha | Rockefeller | Bello | Neiva | Itagüí | Riohacha | Rockefeller |
| 39.90 ± 1.44*[a] | 54.19 ± 1.76*[b] | 79.62 ± 1.79*[c] | 92.40 ± 1.06*[d] | 88.86 ± 1.86*[d] | 21.33 ± 0.76[a] | 27.64 ± 1.09[b] | 44.81 ± 1.64[c] | 75.97 ± 1.44[d] | 32.79 ± 1.17[b] |

**Table S7.** Pairwise strata comparisons between populations by sex was performed using the Log-rank (Mantel-Cox) test. Males and females of each *Ae. aegypti* population and the control Rockefeller were reared at 28 ± 1°C temperature, 80 ± 5% relative humidity, and 12 h light: 12 h dark photoperiod. Chi square ($X^2$). The statistically significant differences (Sig.) were established ($p < 0.05$).

| Sex | Population or control | Bello | | Neiva | | Itagüí | | Riohacha | | Rockefeller | |
|---|---|---|---|---|---|---|---|---|---|---|---|
| | | $X^2$ | Sig. | $X^2$ | Sig. | $X^2$ | Sig. | $X^2$ | Sig. | $X^2$ | Sig. |
| Males | Bello | - | - | 20.59 | <0.001 | 164.60 | <0.001 | 476.87 | <0.001 | 67.99 | <0.001 |
| | Neiva | 20.59 | <0.001 | - | - | 68.90 | <0.001 | 389.12 | <0.001 | 12.05 | 0.001 |
| | Itagüí | 164.60 | <0.001 | 68.90 | <0.001 | - | - | 151.71 | <0.001 | 28.95 | <0.001 |
| | Riohacha | 476.87 | <0.001 | 389.12 | <0.001 | 151.71 | <0.001 | - | - | 314.60 | <0.001 |
| | Rockefeller | 67.99 | <0.001 | 12.05 | 0.001 | 28.95 | <0.001 | 314.60 | <0.001 | - | - |
| Females | Bello | - | - | 40.21 | <0.001 | 261.52 | <0.001 | 435.70 | <0.001 | 336.52 | <0.001 |
| | Neiva | 40.21 | <0.001 | - | - | 90.77 | <0.001 | 186.83 | <0.001 | 170.00 | <0.001 |
| | Itagüí | 261.52 | <0.001 | 90.77 | <0.001 | - | - | 9.10 | 0.003 | 23.50 | <0.001 |
| | Riohacha | 435.70 | <0.001 | 186.83 | <0.001 | 9.10 | 0.003 | - | - | 17.83 | <0.001 |
| | Rockefeller | 336.52 | <0.001 | 170.00 | <0.001 | 23.50 | <0.001 | 17.83 | <0.001 | - | - |

**Table S8.** Life cycle (days) from egg to adult (male and female) of Colombian *Ae. aegypti* from two regions, reared at 21±1°C, 28±1°C and 35±1°C temperatures (Temp), 80±5% relative humidity, and 12 h light: 12 h dark photoperiod. Minimum (Min); Maximum (Max); Standard deviation (SD). Pupation time is the mean development time from L4 to pupae. Time of emergence is the mean development time from pupae to adults (male or female). ANOVA was conducted followed by a Bonferroni post- hoc test to account for multiple comparisons between temperatures at the same age within the same population. Means by age within the same population followed by the same letters are not significantly different ($p > 0.05$).

| | | Bello | | | Neiva | | | Rockefeller | | |
|---|---|---|---|---|---|---|---|---|---|---|
| **Development parameters** | **Temp °C** | **Min** | **Max** | **Mean±SD** | **Min** | **Max** | **Mean±SD** | **Min** | **Max** | **Mean±SD** |
| **Egg to L1 (Eclosion)** | 21 | 1.0 | 12.0 | 2.10 ± 1.57[a] | 1.0 | 42.0 | 2.93 ± 6.90[a] | 1.0 | 39.0 | 3.37 ± 4.10[a] |
| | 28 | 1.0 | 10.0 | 1.22 ± 0.61[b] | 1.0 | 10.0 | 1.39 ± 1.49[b] | 1.0 | 21.0 | 2.19 ± 2.33[b] |
| | 35 | 1.0 | 30.0 | 7.67 ± 3.30[c] | 1.0 | 23.0 | 6.25 ± 5.18[c] | 1.0 | 31.0 | 6.02 ± 4.18[c] |
| **L1+L2 to L3** | 21 | 2.0 | 24.0 | 3.42 ± 2.07[a] | 2.0 | 45.0 | 4.39 ± 7.60[a] | 2.0 | 44.0 | 5.02 ± 5.10[a] |
| | 28 | 2.0 | 11.0 | 2.09 ± 0.63[b] | 2.0 | 10.0 | 3.13 ± 1.43[b] | 2.0 | 25.0 | 4.27 ± 2.70[b] |
| | 35 | 2.0 | 27.0 | 7.94 ± 3.27[c] | 1.0 | 20.0 | 6.29 ± 4.52[c] | 2.0 | 22.0 | 7.19 ± 3.93[c] |
| **L3 to L4** | 21 | 2.0 | 26.0 | 4.54 ± 2.19[a] | 2.0 | 45.0 | 5.33 ± 7.54[a] | 2.0 | 45.0 | 6.07 ± 4.70[a] |
| | 28 | 1.0 | 13.0 | 1.14 ± 0.87[b] | 1.0 | 10.0 | 2.64 ± 1.57[b] | 1.0 | 26.0 | 3.04 ± 2.41[b] |
| | 35 | 1.0 | 26.0 | 6.68 ± 3.37[c] | 1.0 | 18.0 | 5.27 ± 4.36[a] | 1.0 | 20.0 | 5.67 ± 3.82[a] |
| **L1+L2 to L4 (Total larval development time)** | 21 | 4.0 | 35.0 | 7.68 ± 3.05[a] | 4.0 | 90.0 | 9.07 ± 12.91[a] | 4.0 | 89.0 | 10.82 ± 8.70[a] |
| | 28 | 3.0 | 16.0 | 3.17 ± 1.03[b] | 3.0 | 20.0 | 5.70 ± 2.87[b] | 3.0 | 42.0 | 7.18 ± 4.64[b] |
| | 35 | 3.0 | 53.0 | 14.48 ± 6.34[c] | 2.0 | 38.0 | 11.48 ± 8.91[a] | 3.0 | 42.0 | 12.75 ± 7.64[c] |
| **L4 to Pupae (Pupation time)** | 21 | 3.0 | 31.0 | 7.29 ± 2.46[a] | 4.0 | 46.0 | 7.22 ± 7.31[a] | 3.0 | 44.0 | 7.07 ± 4.57[a] |
| | 28 | 2.0 | 11.0 | 3.11 ± 0.73[b] | 2.0 | 10.0 | 6.49 ± 1.49[ab] | 0.0 | 19.0 | 3.90 ± 2.03[b] |
| | 35 | 0.0 | 26.0 | 6.62 ± 3.56[c] | 0.0 | 18.0 | 4.89 ± 4.49[b] | 0.0 | 20.0 | 6.02 ± 3.98[c] |

| | | | | | | | | | | |
|---|---|---|---|---|---|---|---|---|---|---|
| Pupae to Male (Male emergence time) | 21 | 3.0 | 15.0 | 6.21 ± 1.93[a] | 2.0 | 45.0 | 6.65 ± 7.16[a] | 3.0 | 43.0 | 6.37 ± 4.78[a] |
| | 28 | 2.0 | 5.0 | 3.23 ± 0.53[b] | 1.0 | 11.0 | 5.46 ± 1.86[a] | 2.0 | 10.0 | 4.16 ± 1.93[b] |
| | 35 | 1.0 | 26.0 | 7.07 ± 3.46[c] | 1.0 | 16.0 | 4.31 ± 3.21[a] | 0.0 | 20.0 | 6.70 ± 4.07[a] |
| Pupae to Female (Female emergence time) | 21 | 4.0 | 14.0 | 6.85 ± 1.72[a] | 4.0 | 44.0 | 7.39 ± 7.21[a] | 4.0 | 43.0 | 7.29 ± 4.38[a] |
| | 28 | 3.0 | 12.0 | 3.81 ± 0.98[b] | 2.0 | 11.0 | 6.41 ± 1.63[a] | 2.0 | 21.0 | 5.09 ± 2.32[b] |
| | 35 | 2.0 | 27.0 | 7.38 ± 3.59[a] | 2.0 | 19.0 | 7.20 ± 5.20[a] | 1.0 | 16.0 | 6.55 ± 3.98[a] |
| Average age Egg to Male | 21 | 11 | 108.0 | 23.56 ± 10.22 | 11.0 | 223.0 | 26.52 ± 36.51 | 11.0 | 215.0 | 27.9 ± 23.25 |
| | 28 | 8.0 | 50.0 | 10.79 ± 3.37 | 7.0 | 51.0 | 19.11 ± 7.84 | 6.0 | 101.0 | 17.56 ± 11.4 |
| | 35 | 5.0 | 135.0 | 35.98 ± 16.96 | 4.0 | 95.0 | 27.01 ± 21.76 | 4.0 | 113.0 | 31.6 ± 19.98 |
| Average age Egg to Female | 21 | 12.0 | 107.0 | 24.2 ± 10.01 | 13.0 | 222.0 | 27.26 ± 36.56 | 12.0 | 215.0 | 28.82 ± 22.85 |
| | 28 | 9.0 | 57.0 | 11.37 ± 3.82 | 8.0 | 51.0 | 20.06 ± 7.61 | 6.0 | 112.0 | 18.49 ± 11.79 |
| | 35 | 6.0 | 136.0 | 36.29 ± 17.09 | 5.0 | 98.0 | 29.9 ± 23.75 | 5.0 | 109.0 | 31.45 ± 19.89 |

**Table S9.** Egg hatch rate; pupation rate and emergence rate of two Colombian *Ae. aegypti* populations reared at 21±1°C, 28±1°C and 35±1°C temperatures, 80±5% relative humidity, and 12 h light: 12 h dark photoperiod. Minimum (Min); Maximum (Max); Standard deviation (SD) of four to six replicates are shown. ANOVA was conducted followed by a Bonferroni post- hoc test to account for multiple comparisons between temperatures at the same rate within the same population. Means by rate within the same population followed by the same letters are not significantly different ($p > 0.05$).

| | | Bello | | | Neiva | | | Rockefeller | | |
|---|---|---|---|---|---|---|---|---|---|---|
| **Rates** | **Temp °C** | **Min** | **Max** | **Mean±SD** | **Min** | **Max** | **Mean±SD** | **Min** | **Max** | **Mean±SD** |
| **Egg hatch rate** | **21** | **88.0** | **94.0** | **91.2 ± 2.39[a]** | **75.0** | **91.0** | **83.4 ± 7.57[a]** | **87.0** | **99.0** | **92.6 ± 5.03[a]** |
| | **28** | **97.0** | **100.0** | **98.8 ± 1.5[a]** | **87.0** | **92.0** | **89.75 ± 2.22[a]** | **97.0** | **100.0** | **98.8 ± 1.5[a]** |
| | **35** | **57.0** | **81.0** | **68.8 ± 9.2[b]** | **11.0** | **27.0** | **17.4 ± 6.54[b]** | **57.0** | **81.0** | **66.0 ± 11.96[b]** |
| **Pupation rate** | **21** | **74.0** | **87.0** | **79.8 ± 4.9[a]** | **66.0** | **85.0** | **75.2 ± 7.5[a]** | **84.0** | **90.0** | **86.4 ± 2.3[a]** |
| | **28** | **87.0** | **98.0** | **90.3 ± 5.2[a]** | **83.0** | **87.0** | **85.5 ± 1.7[a]** | **90.0** | **97.0** | **94.0 ± 2.8[a]** |
| | **35** | **53.0** | **71.0** | **60.0 ± 6.93[b]** | **9.0** | **20.0** | **13.0 ± 5.15[b]** | **44.0** | **67.0** | **52.6 ± 9.2[b]** |
| **Emergence rate** | **21** | **68.0** | **81.0** | **76.2 ± 4.97[a]** | **66.0** | **85.0** | **73.6 ± 8.1[a]** | **83.0** | **90.0** | **85.6 ± 2.7[a]** |
| | **28** | **83.0** | **87.0** | **84.75 ± 1.71[a]** | **82.0** | **86.0** | **84.3 ± 1.71[a]** | **90.0** | **97.0** | **94.0 ± 2.8[a]** |
| | **35** | **46.0** | **68.0** | **53.4 ± 8.5[b]** | **8.0** | **19.0** | **12.4 ± 4.83[b]** | **42.0** | **65.0** | **51.0 ± 9.1[b]** |

**Table S10.** Age specific life table of 100 cohort eggs in each *Ae. aegypti* population. Mosquitoes were reared at 21±1°C temperature, 80±5% relative humidity, and 12 h light: 12 h dark photoperiod. Mean ± standard deviation (SD) of five replicates are shown. Proportion of age-specific survivorship: *lx*. Proportion of individuals who die during the age interval (*x*) to (*x + 1*): *dx*. Age-specific mortality rate: *qx*. Killing power: *kx*. Average of the probability of survival between two successive ages: *Lx*. Total number of days remaining to live for survivors of the cohort, when it reaches age *x*, until the last member dies at age *m*: *Tx*. Life expectancy, i.e., the mean number of days remaining to the survivors at age *x*: *ex*.

| Parameters | Developmental stage (*x*) | Bello | Neiva | Rockefeller |
|---|---|---|---|---|
| *lx* | Egg | 1.00 ± 0.00 | 1.00 ± 0.00 | 1.00 ± 0.00 |
| | L1+L2 | 0.912 ± 0.024 | 0.834 ± 0.076 | 0.926 ± 0.05 |
| | L3 | 0.85 ± 0.049 | 0.784 ± 0.076 | 0.898 ± 0.039 |
| | L4 | 0.82 ± 0.052 | 0.764 ± 0.076 | 0.882 ± 0.026 |
| | Pupae | 0.798 ± 0.05 | 0.752 ± 0.075 | 0.864 ± 0.023 |
| | Adult | 0.76 ± 0.05 | 0.736 ± 0.081 | 0.856 ± 0.027 |
| *dx* | Egg | 0.088 ± 0.024 | 0.166 ± 0.076 | 0.074 ± 0.050 |
| | L1+L2 | 0.064 ± 0.034 | 0.050 ± 0.026 | 0.028 ± 0.016 |
| | L3 | 0.03 ± 0.005 | 0.020 ± 0.019 | 0.016 ± 0.015 |
| | L4 | 0.022 ± 0.005 | 0.012 ± 0.011 | 0.018 ± 0.008 |
| | Pupae | 0.036 ± 0.043 | 0.016 ± 0.025 | 0.008 ± 0.013 |
| | Adult | 0.76 ± 0.05 | 0.736 ± 0.081 | 0.856 ± 0.027 |
| *qx* | Egg | 0.088 ± 0.024 | 0.166 ± 0.076 | 0.074 ± 0.05 |
| | L1+L2 | 0.070 ± 0.038 | 0.060 ± 0.029 | 0.028 ± 0.016 |
| | L3 | 0.034 ± 0.009 | 0.026 ± 0.026 | 0.016 ± 0.015 |
| | L4 | 0.026 ± 0.005 | 0.014 ± 0.015 | 0.018 ± 0.008 |
| | Pupae | 0.042 ± 0.05 | 0.020 ± 0.034 | 0.008 ± 0.013 |
| | Adult | 1.00 ± 0.00 | 1.00 ± 0.00 | 1.00 ± 0.00 |
| *kx* | Egg | 0.042 ± 0.013 | 0.080 ± 0.038 | 0.034 ± 0.026 |
| | L1+L2 | 0.032 ± 0.019 | 0.028 ± 0.015 | 0.014 ± 0.009 |
| | L3 | 0.018 ± 0.005 | 0.012 ± 0.013 | 0.006 ± 0.005 |
| | L4 | 0.010 ± 0.00 | 0.010 ± 0.007 | 0.008 ± 0.004 |
| | Pupae | 0.022 ± 0.022 | 0.012 ± 0.016 | 0.006 ± 0.009 |
| | Adult | - | - | - |
| *Lx* | Egg | 0.958 ± 0.013 | 0.920 ± 0.038 | 0.966 ± 0.026 |
| | L1+L2 | 0.88 ± 0.033 | 0.812 ± 0.075 | 0.914 ± 0.043 |
| | L3 | 0.84 ± 0.049 | 0.776 ± 0.077 | 0.892 ± 0.034 |
| | L4 | 0.81 ± 0.052 | 0.762 ± 0.076 | 0.876 ± 0.023 |
| | Pupae | 0.78 ± 0.044 | 0.746 ± 0.077 | 0.862 ± 0.023 |
| | Adult | 0.38 ± 0.027 | 0.370 ± 0.043 | 0.430 ± 0.012 |
| *Tx* | Egg | 4.640 ± 0.202 | 4.370 ± 0.371 | 4.926 ± 0.159 |
| | L1+L2 | 3.69 ± 0.194 | 3.456 ± 0.336 | 3.966 ± 0.137 |
| | L3 | 2.81 ± 0.159 | 2.65 ± 0.264 | 3.056 ± 0.093 |
| | L4 | 1.97 ± 0.113 | 1.874 ± 0.188 | 2.164 ± 0.059 |
| | Pupae | 1.16 ± 0.069 | 1.114 ± 0.118 | 1.290 ± 0.037 |
| | Adult | 0.38 ± 0.027 | 0.370 ± 0.043 | 0.430 ± 0.012 |
| *ex* | Egg | 4.640 ± 0.202 | 4.370 ± 0.371 | 4.926 ± 0.159 |
| | L1+L2 | 4.04 ± 0.149 | 4.140 ± 0.129 | 4.284 ± 0.106 |
| | L3 | 3.31 ± 0.044 | 3.374 ± 0.105 | 3.40 ± 0.053 |
| | L4 | 2.40 ± 0.057 | 2.45 ± 0.061 | 2.448 ± 0.013 |
| | Pupae | 1.46 ± 0.05 | 1.480 ± 0.034 | 1.492 ± 0.013 |
| | Adult | 0.50 ± 0.00 | 0.50 ± 0.00 | 0.50 ± 0.00 |

**Table S11.** Age specific life table of 100 cohort eggs in each *Ae. aegypti* population. Mosquitoes were reared at 35±1°C temperature, 80±5% relative humidity, and 12 h light: 12 h dark photoperiod. Mean ± standard deviation (SD) of five replicates are shown. Proportion of age-specific survivorship: *lx*. Proportion of individuals who die during the age interval (*x*) to (*x + 1*): *dx*. Age-specific mortality rate: *qx*. Killing power: *kx*. Average of the probability of survival between two successive ages: *Lx*. Total number of days remaining to live for survivors of the cohort, when it reaches age *x*, until the last member dies at age *m*: *Tx*. Life expectancy, i.e., the mean number of days remaining to the survivors at age *x*: *ex*.

| Parameters | Developmental stage (*x*) | Bello | Neiva | Rockefeller |
|---|---|---|---|---|
| *lx* | Egg | 1.00 ± 0.00 | 1.00 ± 0.00 | 1.00 ± 0.00 |
| | L1+L2 | 0.688 ± 0.092 | 0.174 ± 0.065 | 0.660 ± 0.120 |
| | L3 | 0.622 ± 0.074 | 0.140 ± 0.052 | 0.540 ± 0.088 |
| | L4 | 0.608 ± 0.074 | 0.134 ± 0.054 | 0.528 ± 0.090 |
| | Pupae | 0.60 ± 0.069 | 0.130 ± 0.052 | 0.526 ± 0.092 |
| | Adult | 0.534 ± 0.085 | 0.124 ± 0.048 | 0.510 ± 0.091 |
| *dx* | Egg | 0.31 ± 0.092 | 0.826 ± 0.065 | 0.340 ± 0.120 |
| | L1+L2 | 0.066 ± 0.023 | 0.034 ± 0.018 | 0.120 ± 0.076 |
| | L3 | 0.014 ± 0.015 | 0.006 ± 0.005 | 0.012 ± 0.016 |
| | L4 | 0.008 ± 0.008 | 0.004 ± 0.005 | 0.002 ± 0.005 |
| | Pupae | 0.066 ± 0.030 | 0.006 ± 0.005 | 0.016 ± 0.015 |
| | Adult | 0.534 ± 0.085 | 0.124 ± 0.048 | 0.510 ± 0.091 |
| *qx* | Egg | 0.31 ± 0.092 | 0.826 ± 0.065 | 0.340 ± 0.120 |
| | L1+L2 | 0.094 ± 0.027 | 0.190 ± 0.073 | 0.174 ± 0.098 |
| | L3 | 0.022 ± 0.023 | 0.052 ± 0.050 | 0.022 ± 0.034 |
| | L4 | 0.012 ± 0.013 | 0.028 ± 0.041 | 0.004 ± 0.009 |
| | Pupae | 0.112 ± 0.052 | 0.044 ± 0.046 | 0.032 ± 0.033 |
| | Adult | 1.00 ± 0.00 | 1.00 ± 0.00 | 1.00 ± 0.00 |
| *kx* | Egg | 0.16 ± 0.057 | 0.784 ± 0.158 | 0.184 ± 0.077 |
| | L1+L2 | 0.044 ± 0.015 | 0.092 ± 0.036 | 0.086 ± 0.051 |
| | L3 | 0.012 ± 0.011 | 0.024 ± 0.025 | 0.012 ± 0.016 |
| | L4 | 0.006 ± 0.005 | 0.012 ± 0.018 | 0.002 ± 0.005 |
| | Pupae | 0.052 ± 0.023 | 0.020 ± 0.021 | 0.012 ± 0.013 |
| | Adult | - | - | - |
| *Lx* | Egg | 0.85 ± 0.046 | 0.592 ± 0.033 | 0.834 ± 0.061 |
| | L1+L2 | 0.66 ± 0.081 | 0.160 ± 0.057 | 0.604 ± 0.099 |
| | L3 | 0.62 ± 0.075 | 0.140 ± 0.052 | 0.536 ± 0.091 |
| | L4 | 0.606 ± 0.073 | 0.134 ± 0.054 | 0.528 ± 0.090 |

| | | | | |
|---|---|---|---|---|
| | Pupae | 0.572 ± 0.076 | 0.130 ± 0.052 | 0.520 ± 0.090 |
| | Adult | 0.268 ± 0.042 | 0.064 ± 0.025 | 0.258 ± 0.048 |
| *Tx* | Egg | 3.55 ± 0.39 | 1.202 ± 0.269 | 3.264 ± 0.460 |
| | L1+L2 | 2.71 ± 0.34 | 0.620 ± 0.237 | 2.438 ± 0.410 |
| | L3 | 2.06 ± 0.262 | 0.460 ± 0.180 | 1.834 ± 0.316 |
| | L4 | 1.44 ± 0.186 | 0.326 ± 0.126 | 1.302 ± 0.229 |
| | Pupae | 0.84 ± 0.118 | 0.192 ± 0.073 | 0.776 ± 0.138 |
| | Adult | 0.268 ± 0.042 | 0.064 ± 0.025 | 0.258 ± 0.048 |
| *ex* | Egg | 3.55 ± 0.39 | 1.202 ± 0.269 | 3.264 ± 0.460 |
| | L1+L2 | 3.94 ± 0.13 | 3.532 ± 0.217 | 3.710 ± 0.359 |
| | L3 | 3.298 ± 0.091 | 3.252 ± 0.104 | 3.394 ± 0.134 |
| | L4 | 2.37 ± 0.059 | 2.402 ± 0.070 | 2.460 ± 0.046 |
| | Pupae | 1.39 ± 0.052 | 1.456 ± 0.046 | 1.468 ± 0.033 |
| | Adult | 0.50 ± 0.00 | 0.50 ± 0.00 | 0.50 ± 0.00 |

**Table S12.** Age-specific mortality rate (*qx*) and life expectancy (*ex*) of 100 eggs for each *Ae. aegypti* population. Mosquitoes were reared at 21±1°C, 28±1°C and 35±1°C temperatures, 80±5% relative humidity, and 12 h light: 12 h dark photoperiod. Mean ± standard deviation (SD) of four to six replicates are shown. ANOVA was conducted followed by a Bonferroni post- hoc test to account for multiple comparisons between temperatures for the same parameter within the same population and developmental stage. Means followed by the same letters are not significantly different ($p > 0.05$).

| | | | Bello | Neiva | Rockefeller |
|---|---|---|---|---|---|
| Parameters | Developmental stage (*x*) | Temp °C | Mean±SD | Mean±SD | Mean±SD |
| *qx* | Egg | 21 | 0.088 ± 0.024[a] | 0.166 ± 0.076[a] | 0.074 ± 0.05[a] |
| | | 28 | 0.013 ± 0.015[a] | 0.103 ± 0.022[a] | 0.012 ± 0.015[a] |
| | | 35 | 0.31 ± 0.092[b] | 0.826 ± 0.065[b] | 0.340 ± 0.120[b] |
| | L1+L2 | 21 | 0.070 ± 0.038[ab] | 0.060 ± 0.029[a] | 0.028 ± 0.016[a] |
| | | 28 | 0.028 ± 0.021[a] | 0.015 ± 0.013[a] | 0.025 ± 0.02[a] |
| | | 35 | 0.094 ± 0.027[b] | 0.190 ± 0.073[b] | 0.174 ± 0.098[b] |
| | L3 | 21 | 0.034 ± 0.009[a] | 0.026 ± 0.026[a] | 0.016 ± 0.015[a] |
| | | 28 | 0.025 ± 0.025[a] | 0.018 ± 0.022[a] | 0.013 ± 0.012[a] |
| | | 35 | 0.022 ± 0.023[a] | 0.052 ± 0.05[a] | 0.022 ± 0.034[a] |
| | L4 | 21 | 0.026 ± 0.005[a] | 0.014 ± 0.015[a] | 0.018 ± 0.008[a] |

| | | | | | |
|---|---|---|---|---|---|
| | | 28 | 0.033 ± 0.025[a] | 0.01 ± 0.008[a] | 0.01 ± 0.009[a] |
| | | 35 | 0.012 ± 0.013[a] | 0.028 ± 0.041[a] | 0.004 ± 0.009[a] |
| | Pupae | 21 | 0.042 ± 0.05[a] | 0.020 ± 0.034[a] | 0.008 ± 0.013[a] |
| | | 28 | 0.058 ± 0.064[a] | 0.013 ± 0.013[a] | 0.00 ± 0.00[a] |
| | | 35 | 0.112 ± 0.052[a] | 0.044 ± 0.046[a] | 0.032 ± 0.033[a] |
| *ex* | Egg | 21 | 4.640 ± 0.202[a] | 4.370 ± 0.371[a] | 4.93 ± 0.159[a] |
| | | 28 | 5.133 ± 0.137[a] | 4.843 ± 0.104[a] | 5.282 ± 0.106[a] |
| | | 35 | 3.552 ± 0.386[b] | 1.202 ± 0.269[b] | 3.264 ± 0.460[b] |
| | L1+L2 | 21 | 4.040 ± 0.149[ab] | 4.140 ± 0.129[a] | 4.284 ± 0.106[a] |
| | | 28 | 4.190 ± 0.082[a] | 4.343 ± 0.022[a] | 4.34 ± 0.105[a] |
| | | 35 | 3.942 ± 0.130[b] | 3.532 ± 0.217[b] | 3.710 ± 0.359[b] |
| | L3 | 21 | 3.308 ± 0.044[a] | 3.374 ± 0.105[a] | 3.40 ± 0.053[a] |
| | | 28 | 3.30 ± 0.043[a] | 3.405 ± 0.065[a] | 3.44 ± 0.043[a] |
| | | 35 | 3.30 ± 0.091[a] | 3.252 ± 0.104[a] | 3.394 ± 0.134[a] |
| | L4 | 21 | 2.404 ± 0.057[a] | 2.45 ± 0.061[a] | 2.45 ± 0.013[a] |
| | | 28 | 2.37 ± 0.057[a] | 2.46 ± 0.031[a] | 2.48 ± 0.018[a] |
| | | 35 | 2.37 ± 0.059[a] | 2.402 ± 0.070[a] | 2.46 ± 0.046[a] |
| | Pupae | 21 | 1.46 ± 0.05[a] | 1.480 ± 0.034[a] | 1.492 ± 0.013[a] |
| | | 28 | 1.44 ± 0.064[a] | 1.49 ± 0.013[a] | 1.50 ± 0.00[a] |
| | | 35 | 1.39 ± 0.052[a] | 1.46 ± 0.046[a] | 1.47 ± 0.033[a] |
